# Comparison between FEBio and Abaqus for biphasic contact problems

**DOI:** 10.1177/0954411913483537

**Published:** 2013-09

**Authors:** Qingen Meng, Zhongmin Jin, John Fisher, Ruth Wilcox

**Affiliations:** 1 Institute of Medical and Biological Engineering, School of Mechanical Engineering, University of Leeds, Leeds, UK; 2 School of Mechanical Engineering, Xi’an Jiaotong University, Xi’an, China

**Keywords:** Articular cartilage, biphasic model, finite element, Abaqus, FEBio

## Abstract

Articular cartilage plays an important role in the function of diarthrodial joints. Computational methods have been used to study the biphasic mechanics of cartilage, and Abaqus has been one of the most widely used commercial software packages for this purpose. A newly developed open-source finite element solver, FEBio, has been developed specifically for biomechanical applications. The aim of this study was to undertake a direct comparison between FEBio and Abaqus for some practical contact problems involving cartilage. Three model types, representing a porous flat-ended indentation test, a spherical-ended indentation test, and a conceptual natural joint contact model, were compared. In addition, a parameter sensitivity study was also performed for the spherical-ended indentation test to investigate the effects of changes in the input material properties on the model outputs, using both FEBio and Abaqus. Excellent agreement was found between FEBio and Abaqus for all of the model types and across the range of material properties that were investigated.

## Introduction

Articular cartilage (AC) plays an important role in the function of diarthrodial joints. It helps to distribute the loads between opposing bones over a large contact area and minimize the contact stress. The biphasic nature of AC and in particular load carriage by the fluid phase provides a bearing surface with low friction and wear over a life span. It is important to understand the mechanical behavior of cartilage in order to develop effective treatments for damaged or diseased joints. It is generally accepted that for a correct description of the mechanical behavior of AC, at least a biphasic model^1^ should be used. Analytical solutions can be found for only a limited number of idealized biphasic problems, such as confined and unconfined compression tests. For problems with complex geometry and under realistic conditions, it is necessary to use numerical approximation techniques, such as the finite element method.

Abaqus (Dassault Systèmes, Waltham, MA, USA) is a commonly used commercial finite element program. Finite element models that include cartilage components can be solved using the soil consolidation theory within Abaqus, and it has been one of the most widely used programs to study the biphasic mechanics of cartilage since the 1990s. Studies that employ Abaqus have been extended from those that evaluate its feasibility to analyze biphasic soft tissues^2,3^ to practical applications where cartilage is simulated within a realistic problem.^4–9^ Recently, a freely available, open-source nonlinear finite element solver, FEBio (Musculoskeletal Research Laboratories, University of Utah, Salt Lake City, UT, USA), was developed specifically for biomechanical applications.^10,11^ Porous media problems, such as the biphasic mechanics of cartilage, can be solved by FEBio using the biphasic material model embedded within the code. A finite element contact implementation for biphasic materials is available in the code. This implementation is able to accommodate finite deformation and large sliding.^11^

The comparison between different codes is important for verification, especially for practical problems where no analytical solutions exist. In biomechanical analyses, it is common practice to compare the solutions of the same problem produced by different codes, especially for newly developed software or new applications of existing algorithms. Previously, comparisons have been made between other software packages for a confined compression problem to examine the feasibility of available soil mechanic codes to analyze biphasic tissue mechanics.^12^ Comparisons have also been made between the commercial package COMSOL Multiphysics (COMSOL, Inc., Burlington, MA, USA) and previous codes to validate the implementation of augmented Lagrangian method in COMSOL Multiphysics.^13^ The direct comparison between Abaqus and FEBio is particularly important since FEBio has been developed recently, and Abaqus is one of the most established finite element packages in the field. A wide range of comparative tests have been undertaken to examine the performance of FEBio relative to Abaqus for different material models, geometry, and conditions.^10^ However, no biphasic problems have been included in these comparisons. Therefore, the aim of this study was to undertake a direct comparison between FEBio and Abaqus for three practical biphasic problems involving cartilage.

## Methods

Three cartilage models were solved using both Abaqus (Version 6.9-EF1) and FEBio (Version 1.5.0). The first two represented standard experimental characterization procedures using a porous flat-ended indentation test and a solid spherical-ended indentation test. The third represented contact between two conforming cartilage surfaces as is present in the diarthrodial joints. Since both displacement and load controls are commonly employed in such problems, displacement control was used in the porous flat-ended indentation test and load control was applied to the spherical-ended indentation test.

**Figure 1.**
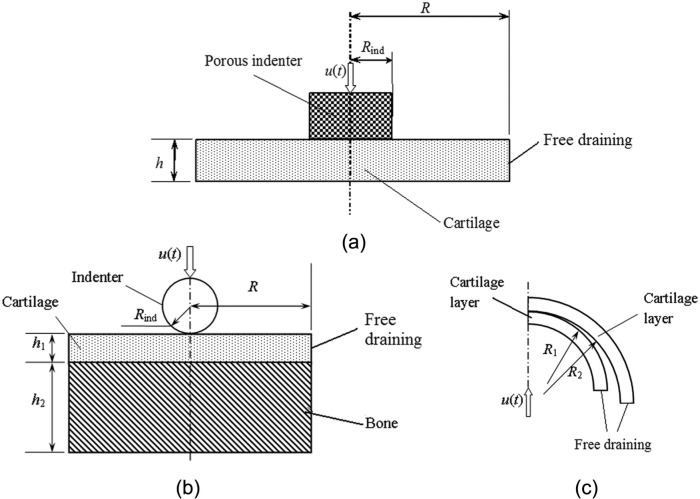
Schematic diagrams of (a) the porous flat-ended cylindrical indentation test, (b) the spherical-ended indentation test, and (c) the joint contact model.

The flat-ended indentation model (Figure 1(a)) comprised a cylindrical cartilage layer (*R* = 15 mm, *h* = 3.0 mm) indented normal to the cartilage surface by a flat-ended porous cylinder (*R*_ind_ = 3 mm) with frictionless contact. The base of the cartilage was fully constrained to simulate an ideal bond between the cartilage and the bone. A given displacement was applied to the indenter over a 2 s period, and this was then held for a further 1200 s. Two displacements were investigated: one of 0.15 mm, representing a compression ratio (*e*) of 5%, and the other of 0.54 mm, representing a compression ratio of 18%. The top and the side surfaces of cartilage were free-draining. No flow was allowed at the bottom surface of the cartilage.

The spherical-ended indentation model (Figure 1(b)) comprised a cylindrical cartilage layer bonded to impermeable bone, compressed by a solid spherical-ended indenter. The thicknesses of the cartilage (*h*_1_) and the bone (*h*_2_) were 2 and 5 mm, respectively; the radii of the cartilage pin (*R*) and indenter (*R*_ind_) were 4.5 and 1.5 mm, respectively. The bottom surface of the bone was fully constrained. A ramp load was applied to the indenter over a period of 2 s and then held for a further 1200 s. Two loads were investigated: a light load of 0.22 N, representing an approximate compression ratio of 10%, and a heavier load of 0.7 N, representing an approximate compression ratio of 20%. The side surface of the cartilage pin was free-draining. A contact-dependent surface fluid flow boundary condition was applied to the surface of the cartilage pin in contact with the indenter because, due to the impermeable nature of the indenter, there would be no fluid flow normal to the surface in the contact region, while the fluid would flow freely in noncontacting regions.^14,15^

The conceptual joint contact model comprised two conforming cartilage layers (Figure 1(c)). Both cartilage layers were 2 mm thick. The radii of the curvature of the contact surfaces were *R*_1_ = 25.5 mm and *R*_2_ = 26 mm. The upper surface of the top cartilage layer was fully constrained. A vertical displacement was applied to the lower surface of the lower cartilage over 10 s and held constantly for a further 300 s. Two displacements of 0.04 and 0.2 mm were investigated, representing compression ratios of 1% and 5%, respectively. The side surfaces of the cartilage layers were free-draining. Since the bottom surface of the lower cartilage layer and the top surface of the upper cartilage layer were assumed to be bonded on the bone, no fluid flow was allowed from these surfaces. A contact-dependent surface fluid flow boundary condition was applied to the contacting surfaces where flow was dependent on the fluid pressure difference across the interface in the contacting region, and there was a free-flow condition in the noncontacting regions.^11,15–17^

In all the above-mentioned models, Young’s modulus and Poisson’s ratio of the cartilage were 0.54 MPa and 0, respectively.^18,19^ The void ratio of cartilage was assumed to be 4.0 (solidity of 0.2). Although the permeability of cartilage is believed to be deformation dependent,^20,21^ it was assumed to be a constant of 0.004 mm^4^/N s, since the focus of this study was to compare FEBio and Abaqus under the same conditions. Where bone was included in the model, it was assigned an elastic modulus of 2.0 GPa and Poisson’s ratio of 0.2. The indenters were considered as rigid.

A parametric sensitivity study was also performed for the spherical-ended indentation model (0.22 N load case), using both Abaqus and FEBio, to investigate the agreement between FEBio and Abaqus over a wider range of parameter values, as well as the effects of Young’s modulus and permeability on the spherical-ended indentation model. In the sensitivity study, one input parameter in the model was changed while the others were kept at the baseline values. Both Young’s modulus and permeability of the cartilage were changed by ±10% and ±50% from their original values.

Since all the problems considered in this study were axisymmetric, for computational efficiency and to avoid the convergence difficulties of solving three-dimensional (3D) biphasic models in Abaqus, axisymmetric models were developed and solved in Abaqus. In FEBio, where there was no axisymmetric stress state option in Version 1.5.0, a quarter of each model was solved by applying appropriate symmetry boundary conditions. The mesh densities adopted for each model were determined after a mesh convergence study. The changes in the peak fluid pressure caused by doubling the meshes used in the present study were less than 1% for all models. The number of elements for the cartilage in each model is summarized in Table 1.

**Table 1.** Numbers of element for the cartilage of each model.

		Abaqus	FEBio
Porous flat-ended indentation model		17,280	77,952
Spherical-ended indentation model		2000	45,375
Joint contact model	Upper cartilage layer	2880	8000
	Lower cartilage layer	2880	8000

In the Abaqus models, the cartilage region was discretized with CAX4P (four-node bilinear displacement and pore pressure) elements. Where the bone was included (the spherical-ended indentation model), it was discretized with CAX4 (four-node bilinear stress/displacement axisymmetric) elements. The *soils, consolidation* analysis procedure was used to solve the models. The cartilage permeability was converted into that required for the poroelastic model by multiplying it by the volume weight of the interstitial fluid.^3^ Automatic time incrementation was employed for each model by specifying an appropriate value for *UTOL* that was less than 8% of the maximum pore pressure.^2^ Linear elastic material was used for the solid phase of the cartilage. The *NLGEOM* parameter was used to account for the finite deformation so that comparisons could be made with FEBio, which is based on finite deformation theory.^10,11^*Surface-to-surface* contact discretization and a *finite sliding* tracking approach were used for all the models. Therefore, by default, a penalty method was used as the constraint enforcement method. For all the models, flow was not permitted across the axis of symmetry. Free-draining boundary conditions were specified by giving a zero value to the pore pressure of the nodes on these surfaces. The contact-dependent flow boundary conditions were achieved using user-developed subroutines.^15–16^ To examine the effect of the contact-dependency subroutine, the spherical-ended indentation model and the conceptual joint contact model were also solved using the default fluid flow boundary conditions in Abaqus (i.e. by removing the applied subroutine, such that the enforced relation between fluid flow and fluid pressure difference at the contact area and the free-draining outside the contact region were eliminated).

Before the 3D finite element models were solved using FEBio, solid models were created and meshed using Preview 1.7 (Musculoskeletal Research Laboratories) or NX-IDEAS 6.1 (Siemens Product Lifecycle Management (PLM) Software Inc., Plano, TX, USA). Eight-node hexahedral or six-node hexahedral solid elements were used to discretize the models. *Neo-Hookean* was used as the solid-phase material of the cartilage to account for the finite deformation. It should be noted that prior to this study, a uniaxial analysis on the elastic materials in FEBio and Abaqus was performed. The results showed that under finite deformation, the *Neo-Hookean* material in FEBio and the linear elastic material in Abaqus predicted almost identical stress values for applied strains up to 20% when the material properties used in this study were applied. The *biphasic* analysis step was used to solve these biphasic contact problems. The *sliding2* implementation, which by default uses *facet-to-facet* discretization and takes large sliding into account, was defined as the contact interface. The penalty method was used to enforce the contact constraints. The *auto-penalty* was applied for all models to calculate a suitable initial value for the penalty factor. Flow was prevented from the symmetric surfaces of cartilage of each model. Similar to Abaqus, free-draining boundary conditions were specified by giving zero pore pressure. The contact-dependent surface fluid flow boundary conditions were satisfied automatically.^11^

The average difference, *R_v_*, in the examined variables was used to estimate the agreement between FEBio and Abaqus, which was defined as

(1) $$R_{v}=\frac{1}{n}\sum_{i=1}^{n}\left(\frac{\left|v_{i}^{\text{Abaqus}}-v_{i}^{\text{FEBio}}\right|}{v_{i}^{\text{FEBio}}}\right)$$

where *v_i_* is the examined variable, such as reaction force, fluid pressure, or displacement, at time step *i*, and *n* is the total number of time steps.

## Results

**Figure 2.**
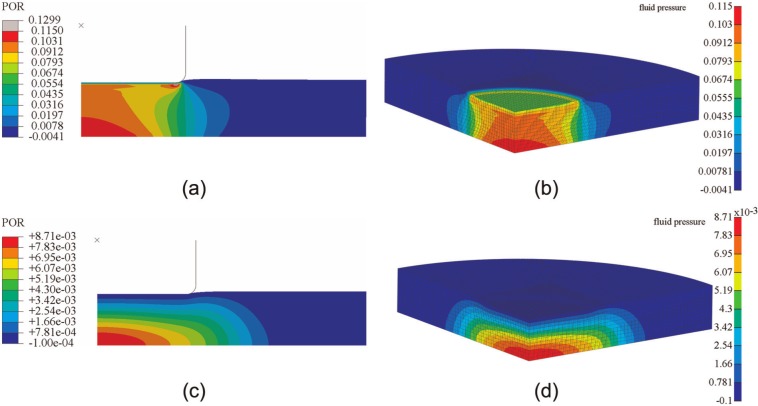
Fluid pressure distribution in the cartilage of the porous flat-ended indentation model subjected to a displacement of 0.15 mm, at 2 s, obtained by (a) Abaqus and (b) FEBio and for the same model at 1200 s, obtained by (c) Abaqus and (d) FEBio. POR stands for the pore pressure in Abaqus

**Figure 3.**
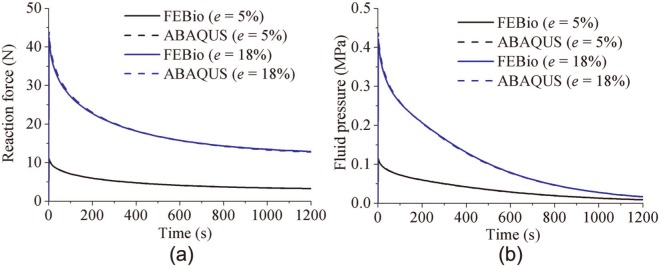
Comparison between FEBio and Abaqus of (a) the reaction force on the indenter and (b) the fluid pressure at the bottom of the cartilage (under the contact center) for the porous flat-ended model under different displacements.

For the porous flat-ended indenter model, the predicted fluid pressure distribution at 2 and 1200 s was found to be very similar in Abaqus and FEBio, as shown in Figure 2, although there were some minor local differences. For both displacements, the reaction force acting on the indenter and the fluid pressure in the cartilage at a reference node (under the contact center and at the bottom of the cartilage pin) predicted by Abaqus and FEBio were nearly identical (Figure 3). The average differences between FEBio and Abaqus in the reaction force for the smaller and larger deformation cases were 0.3% and 1.0%, respectively. The corresponding average differences in the fluid pressure were 0.7% and 1.0%.

**Figure 4.**
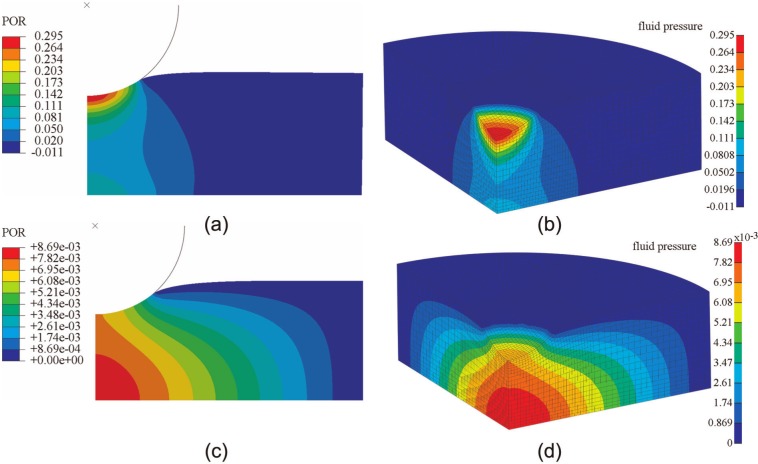
Fluid pressure distribution in the cartilage of the spherical-ended indentation model subjected to a load of 0.7 N, at 2 s, obtained by (a) Abaqus and (b) FEBio and for the same model at 1200 s, obtained by (c) Abaqus and (d) FEBio.

**Figure 5.**
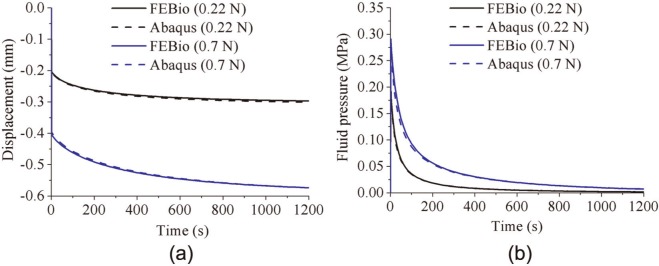
Comparison between FEBio and Abaqus of (a) the displacement of the indenter and (b) the fluid pressure at the contact center for the spherical-ended model under different forces.

FEBio and Abaqus produced similar results for the spherical-ended indentation model. At 2 and 1200 s, the fluid pressure distributions predicted by the two codes were very similar (Figure 4). For both smaller and larger deformation cases, both the vertical displacement of the indenter and the fluid pressure at the reference node (at the contact center of the cartilage pin) predicted by Abaqus and FEBio were very close (Figure 5). The average differences between FEBio and Abaqus in the vertical displacement for the smaller and larger deformation were 1.1% and 1.4%, respectively. The corresponding average differences in the fluid pressure at the reference node were 2.5% and 2.3%, respectively.

**Figure 6.**
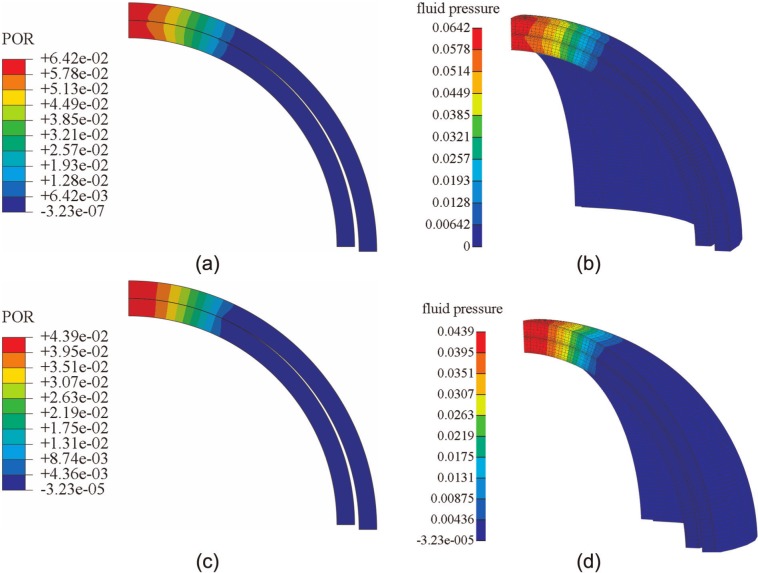
Fluid pressure distribution for the joint contact model subjected to displacement of 0.04 mm, at 10 s, obtained by (a) Abaqus and (b) FEBio and for the same model, at 310 s, obtained by (c) Abaqus and (d) FEBio.

**Figure 7.**
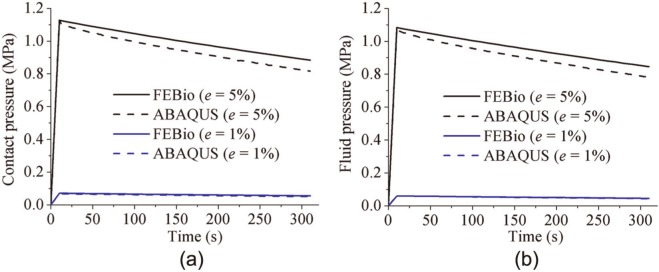
Comparison between FEBio and Abaqus of (a) the fluid pressure and (b) the contact pressure at the contact center of the joint contact model under different displacements.

FEBio and Abaqus also produced similar results for the conceptual joint contact model. The fluid pressure distributions predicted by FEBio and Abaqus closely matched at both 10 and 300 s (Figure 6). For both the 0.04 and 0.2 mm displacement cases, the fluid pressure and the contact pressure at the reference node (at the contact center) predicted by Abaqus and FEBio were very similar (Figure 7). The average differences in fluid pressure between FEBio and Abaqus for cases of *e* = 1% and 5% were 2.0% and 5.4%, respectively. The corresponding average differences in the contact pressure were 7.0% and 7.6%, respectively.

**Figure 8.**
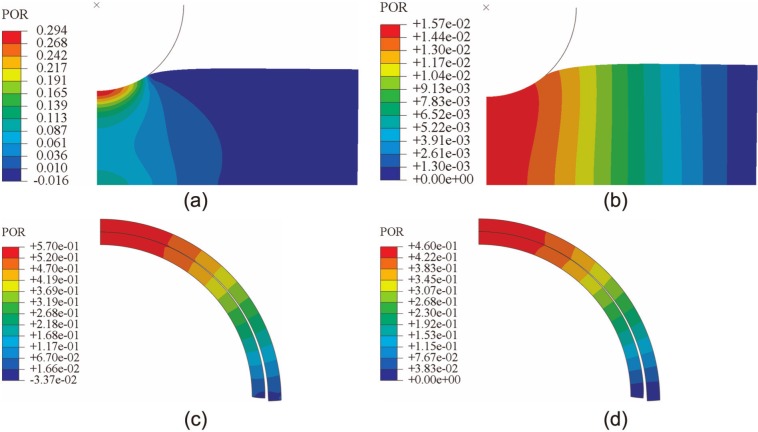
Fluid pressure distributions produced by Abaqus when the contact-dependent surface fluid flow boundary conditions were not specified, showing the results for the spherical-ended indentation model at (a) 2 and (b) 1200 s and for the joint contact model at (c) 10 and (d) 310 s.

The fluid pressure distributions of the models solved using Abaqus with the default fluid flow boundary conditions (i.e. without specifying the contact-dependent surface fluid flow boundary conditions using subroutines) are shown in Figure 8. Notably, fluid pressurization was seen to occur at noncontacting areas. The fluid pressure distribution of the spherical-ended indentation model at 1200 s was clearly different from those shown in Figure 4(c) and (d). The fluid pressure distributions of the joint contact model at 10 and 310 s were also different from those shown in Figure 6.

**Figure 9.**
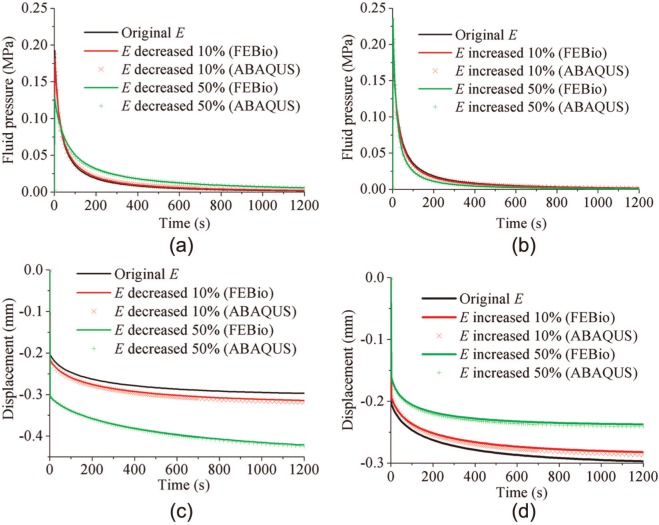
Effect of changes in the elastic modulus of the cartilage on (a, b) the fluid pressure and (c, d) the vertical displacement of the indenter for the spherical-ended indentation test, calculated by both FEBio and Abaqus.

**Figure 10.**
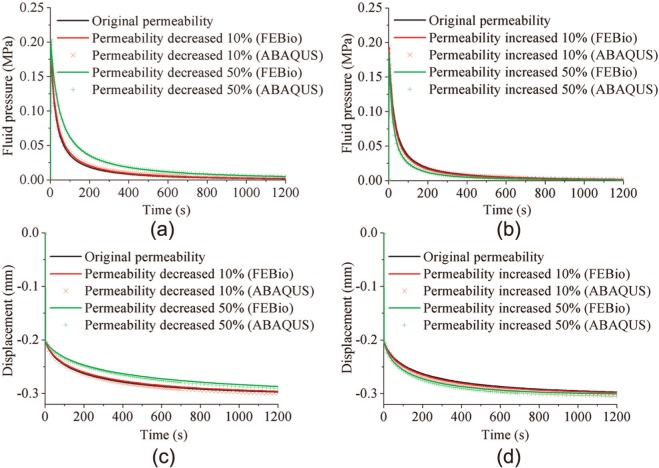
Effect of changes in the permeability on (a, b) the fluid pressure and (c, d) the vertical displacement of the indenter for the spherical-ended indentation test, calculated by both FEBio and Abaqus.

For the wide range of material properties investigated for the spherical-ended indentation model, FEBio and Abaqus agreed well (Figures 9 and 10). The maximum average differences between Abaqus and FEBio in the fluid pressure for the cases presented in Figures 9 and 10 were 3.1%, and the maximum average differences in the vertical displacement were 1.4%. The variation in Young’s modulus of the cartilage had three notable effects on its mechanical behavior. A smaller Young’s modulus reduced the peak fluid pressure, prolonged the time to approach the equilibrium condition, and produced a larger equilibrium deformation (Figure 9). The model predictions were less sensitive to the variation in the permeability of the cartilage. A smaller permeability slightly increased the peak fluid pressure and took longer time to reduce the fluid pressure to zero (Figure 10). The differences in the peak fluid pressure (at 2 s) and the final vertical displacement, along with the maximum differences in the fluid pressure of the reference node (at the contact center of the cartilage pin) and the displacement of the indenter during the whole creep process, caused by all the investigated changes in Young’s modulus and permeability of cartilage, are summarized in Table 2. In all cases, the 50% decrease in Young’s modulus and the 50% decrease in the permeability caused the maximum differences in the four criteria, as shown in Table 2.

**Table 2.** Differences in the fluid pressure and vertical displacement of the indenter for the spherical-ended indentation model caused by the changes in Young’s modulus and permeability of the cartilage.

	Difference in the peak fluid pressure (at 2 s)	Maximum difference in the fluid pressure of the reference node through the whole creep period	Difference in the final vertical displacement of indenter	Maximum difference in vertical displacement of indenter through the whole creep period
*E* decreased 10%	5.4% ↓	23.7% ↑	5.8% ↑	6.1% ↑
*E* increased 10%	4.6% ↑	22.4% ↓	4.9% ↓	5.4% ↓
*E* decreased 50%	31.8% ↓	259.6% ↑	40.0% ↑	48.0% ↑
*E* increased 50%	22.4% ↑	65.6% ↓	20.0% ↓	20.9% ↓
Permeability decreased 10%	1.3% ↑	22.1% ↑	0.4% ↓	1.0% ↓
Permeability increased 10%	1.2% ↓	17.4% ↓	0.3% ↑	0.9% ↑
Permeability decreased 50%	7.6% ↑	210.0% ↑	3.5% ↓	6.2% ↓
Permeability increased 50%	5.5% ↓	59.4% ↓	1.2% ↑	3.8% ↑

↑ denotes increase and ↓ denotes decrease, compared with original values.

## Discussion

Abaqus is a commonly used software package. Biphasic models are solved in Abaqus using the soil consolidation theory.^2,21^ FEBio is based on biphasic theory that is derived from mixture theory of porous media.^10^ When the solid and fluid phases are assumed intrinsically incompressible, the fluid is assumed as inviscid and the inertial effects are neglected, as considered for cartilage, and the soil consolidation theory and biphasic theory are equivalent.^22,23^ They are therefore expected to produce similar solutions for the same biphasic problem. However, few direct comparisons have been performed for cartilage biphasic problems in the literature, particularly for practical applications or more complex geometry. The present study provided evidence for this theoretical agreement by solving the same models using both FEBio and Abaqus.

In the present study, three practical biphasic models, the porous flat-ended indentation test, the spherical-ended indentation test, and the cartilage-on-cartilage model, were compared under different levels of deformation. Moreover, a wide range of material properties was also considered for the spherical-ended indentation test. For all the cases investigated, FEBio and Abaqus produced similar solutions (Figures 2–7, 9, and 10).

It should be noted that there are also some differences in the contact interface methods employed by the two software packages. For example, for the rigid-on-cartilage models, if the contact-dependent surface fluid flow boundary conditions are not specified in Abaqus (Version 6.9-EF1) models, the contacting surface of cartilage is sealed by default.^21,24^ For cartilage-on-cartilage models, in the transient analysis of Abaqus (Version 6.9-EF1), by default, the fluid flowing into the interface is balanced with the rate of separation of the two surfaces (i.e. fluid also flows into the contacting surfaces at the noncontacting regions).^21^ Therefore, if the contact-dependent surface fluid flow boundary conditions were not satisfied using user-written subroutines for the spherical-ended indentation model and the conceptual joint contact model, Abaqus (Version 6.9-EF1) will produce different fluid pressure distributions from FEBio, as shown in Figure 8. This is an important advantage of FEBio over Abaqus in terms of solving biphasic contact problems, since FEBio is able to enforce these conditions automatically.

It should be noted that in later versions of Abaqus (e.g. Versions 9.11 and 9.12), the keyword *Contact Permeability is able to enhance the control over the pore fluid contact properties across a contact interface. However, this keyword was not suitable for the spherical-ended indentation model because it is only applicable when pore pressure degrees of freedom are present on both sides of a contact interface; otherwise, the surfaces are treated as impermeable as in earlier versions.^25^ For cartilage-on-cartilage models, *Contact Permeability is able to control the distance beyond which no fluid flow occurs. However, the requirement for a free-draining condition outside the contact region would still require a subroutine.

The fluid pressure distribution profiles obtained in the present study were also consistent with previous studies for both the porous flat-ended indentation model (Figure 2)^13,17,26^ and the spherical-ended indentation model (Figure 4).^15,17^ The profiles of the fluid pressure distribution of the conceptual joint contact (Figure 6) were consistent with a previous conformal cartilage-on-cartilage model.^13^

From the parameter sensitivity studies using the spherical-ended indentation model, it was found that there was little difference between the results obtained from the two software codes over a larger range of input values. In addition to this comparison, the parameter sensitivity study also provided insight into how the salient parameters of cartilage affect its mechanics. This is particularly important if the code is used to determine the mechanical properties of cartilage. These properties have been derived previously from experimental indentation tests using a computational model and iteratively altering the value of the cartilage properties until the predicted deformation–time curve matches that of the experimental test.^18,27,26^ It is clear from the present study that such a deformation–time curve has a low sensitivity to the value of permeability. The maximum difference in the vertical displacement of the indenter was only 6.2%, compared to the initial curve, when the permeability decreased by 50%. However, it was sensitive to Young’s modulus of the cartilage, with a 50% decrease in modulus causing a maximum 48.0% change in the displacement. The peak fluid pressure was also less sensitive to the permeability than to Young’s modulus of the cartilage, with the differences caused by a 50% decrease in these parameters being 7.6% and 31.8%, respectively. However, it should be noted that more generally, both Young’s modulus and permeability played an important role in the fluid pressure, during the whole cartilage creep period. The maximum differences caused by the 50% decrease in the permeability and Young’s modulus were 210.0% and 259.6%, respectively. The understanding of the effect of these parameters is important for future studies to gauge the level of accuracy needed in assigning the properties to computational models.

There are some limitations in this study. First, the fluid and contact pressures achieved in this study were quite low, compared with expected physiological values. Second, only three practical models were presented and compared in this study. These models were relatively simple and did not include factors such as the collagen fiber directions^29^ and the nonlinearity of the permeability.^19^ However, they provide a baseline set of data that provides confidence that the two software packages are comparable, and further tests could now be undertaken to examine additional complexity in the material models.

## Conclusion

FEBio and Abaqus were compared through solving three practical contact problems involving cartilage: the porous flat-ended indentation test, spherical-ended indentation test, and conceptual joint contact model. For different loading conditions, and for a range of different material properties, FEBio and Abaqus produced similar results for these models. However, if user-written subroutines were not used for Abaqus (Version 6.9-EF1), then Abaqus (Version 6.9-EF1) produced different results for problems involving contact-dependent surface fluid flow boundary conditions.
